# Ultra‐Fast Recyclable and Value‐Added Desulfation Method for Spent Lead Paste via Dual Intensification Processes

**DOI:** 10.1002/advs.202304863

**Published:** 2023-10-22

**Authors:** Lulu Chai, Zhiyu Li, Keyu Wang, Xiaowei Liu, Shaozhen Dai, Xiaoguang Liu, Yanzhi Sun, Junqing Pan

**Affiliations:** ^1^ State Key Laboratory of Chemical Resource Engineering College of Chemistry Beijing University of Chemical Technology Beijing 100029 China; ^2^ Chilwee Power Group Changxing Zhejiang 313100 China

**Keywords:** desulfation process, intensification process, rotating liquid film reactor, spent lead paste recovery, ultra‐rapid cleaning process

## Abstract

The new low‐cost clean pre‐desulfation technology is very important in pyrometallurgy and hydrometallurgy. However, traditional reactors have low space‐time yield and desulfation rate, resulting in high energy consumption and SO_2_ emissions in the industrial desulfation processes. Herein, dual rotating liquid film reactors (RLFRs) and lime are proposed to construct a recyclable, ultra‐fast, and value‐added desulfation method. Parameter optimization and kinetic calculations prove that the above reactions are controlled by internal diffusion, revealing that RLFR promotes the mass transfer and reaction rate. The new process greatly shortens the desulfation time of lead paste from 40 min to 10 s with a high desulfation rate of 99.7%, and the sulfation time of lime from 30 min to 30 s with a sulfation rate of 98.6% with a net profit of 55.99 ¥/ton by cost accounting. Moreover, ten batches of continuous scale‐up experiments demonstrate the stability of processes, the desulfation and sulfation rates are kept at 99.7% and 98.2%, which greatly reduces the emissions of waste desulfate liquor. This work provides a new universal strategy for a sustainable, low‐cost, and clean desulfation method of waste resources to achieve technical and economic feasibility.

## Graphical abstract

This work proposes a new ultra‐fast recyclable intensification desulfation process of lead paste and a mother liquor regenerated by lime via dual rotating liquid film reactors to grind the surface of particles and enhance the mass transfer process, which achieves the desulfation rate and conversion rate of 99.7% and 98.6% in 10 and 30 s, respectively.

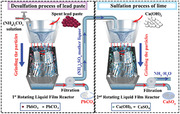

## 1 Introduction

Power batteries are considered the top priority for new energy vehicles to achieve the carbon neutrality goal.^[^ ^1^, ^2^ ^]^ However, the limited cycles of batteries are inevitable to scrap huge quantities of batteries, which contain precious metals such as lead, nickel, cobalt, and lithium. Lead is an important nonferrous heavy metal widely used in lead‐acid batteries (LABs), chemical equipment, nuclear industries, and cable communication.^[^ ^3^, ^4^ ^]^ The emission of heavy metal lead in LABs causes severe damage to human health in the brain and nervous system. With the rapid sustainable development of the energy storage industry in emerging economies, 8.3 million tons of refined lead are used to manufacture the production of LABs, which accounts for over 80% of the total global refined lead production in 2022. From the point of view of resource consumption or the environment, a large number of spent LABs for effective and rational recycling are the focus of attention to achieve the sustainable development of lead resources and reduce environmental pollution.^[^ ^5^, ^6^, ^7^, ^8^ ^]^ Spent lead paste, formed by the active material after long‐term charge and discharge, is the high‐quality lead‐containing resource recovered from spent LABs, which mainly contains PbSO_4_ (50–60%), PbO_2_ (15–35%), PbO (5–10%), Pb (2–5%), and other impurities components (1%) such as Fe, Ba, Al, Sn, and Sb.^[^ ^9^, ^10^ ^]^ Among components of spent lead paste, PbSO_4_ is the main leady compound and is most difficult to be dissolved in water and acid.^[^ ^11^ ^]^ It is also thermodynamic stable, even under a high temperature of 1300 °C. The decomposition of PbSO_4_ phases leads to the emission of SO_2_ and lead particulates. The desulfation process in pyrometallurgy generates emissions of SO_2_ and lead dust and requires a lot of energy. The desulfation process in hydrometallurgy has technical bottlenecks such as expensive desulfurizers, low leaching rate, long reaction time, and high disposal cost of liquid waste treatment.^[^ ^12^, ^13^, ^14^ ^]^ Therefore, from the point of view of resource consumption or environmental protection, the pursuit of a clean, energy‐saving, value‐added, highly efficient, and ultra‐fast pre‐desulfation process of lead paste is very important in the smelting process and hydrometallurgy.

The reported new desulfation technologies include new desulfation agents, desulfation equipment, and related technologies.^[^ ^15^ ^]^ Most of the reported desulfurizers, such as Na_2_CO_3_,^[^ ^16^ ^]^ K_2_CO_3_,^[^ ^17^ ^]^ CaCO_3_,^[^ ^18^ ^]^ (NH_4_)_2_CO_3_,^[^ ^19^, ^20^ ^]^ NH_4_HCO_3_,^[^ ^21^ ^]^ and NaOH,^[^ ^22^, ^23^ ^]^ can convert PbSO_4_ into PbCO_3_ or Pb(OH)_2_ with a dissatisfactory desulfation rate of 95–99% and a long desulfation time of 10–120 min, which is because the solubility of insoluble PbCO_3_ is much smaller than that of PbSO_4_ in the spontaneous solid–liquid phase reaction, resulting in a sluggish kinetics process, reaction time, and conversion rate. Recently, Yang et al.^[^ ^24^ ^]^ used NaOH as a desulfurizer to desulfurize in the hydrothermal reactor with a high desulfation rate of 99.46% for 1 h. Liu et al.^[^ ^21^ ^]^ compared the desulfation effect of Na_2_CO_3_, (NH_4_)_2_CO_3_, and NH_4_HCO_3_ on the spent lead paste, and found that the type of desulfurizer displayed a little effect on desulfation time and rate, and selected 1 h as the optimal operation time. Expensive desulfurizers of NaOH and Na_2_CO_3_ with subsequent evaporation cost of sulfate mother liquor, and slow desulfation speed greatly increase the cost of desulfation, hindering the industrialization application of pre‐desulfation technology. In addition, the Junqing Pan group reported a lime ex situ nucleation + ammonia‐CO_2_ cycle closed loop desulfation process to reduce the cost of desulfurizer.^[^ ^25^ ^]^ The ex situ nucleation process greatly improves the utilization rate of the lime conversion process, achieving a desulfation rate of 99.4% within 60 min. In terms of reactor modification to improve the reaction speed,^[^ ^26^ ^]^ Zhang et al.^[^ ^27^ ^]^ reported the “surface update” process to significantly short the desulfation time by quickly removing the desulfation product from the surface of PbSO_4_ particles with the help of mechanical force, which achieved an extremely low PbSO_4_ residue of 0.5% and ultra‐fast desulfation time of 20 min compared to the traditional process (2% and 60 min). Ma et al.^[^ ^28^ ^]^ proposed a forced surface renewal mechanism for adaptive surface grinding based on magnetic levitation, which greatly improved reaction efficiency and mass transfer speed, resulting in an increased desulfation rate of 97% at 3 h. Ning et al.^[^ ^29^ ^]^ used the rotating packed bed as the reactor for enhanced desulfation of spent lead paste, achieving a high desulfation rate of 97.2% in a discontinuous desulfation process.

The major reasons for the low desulfation rate are the following problems: 1) Most stirred reactors own limited space and low shear force, leading to a low conversion rate and increasing energy consumption due to the unreacted raw materials to be covered by the generated product coating layer. 2) Most stirred reactors have long reaction times and low space‐time yield due to low kinetic rate. 3) Most of the stirred reactors possess a discontinuous reaction process, resulting in low production efficiency and inconsistent products of different batches. Therefore, it is urgent to design and adopt a new highly efficient intensification reactor and inexpensive desulfation agents to construct a fast and continuous desulfation process to promote the future industrial application of pre‐desulfation.

**Scheme 1:**
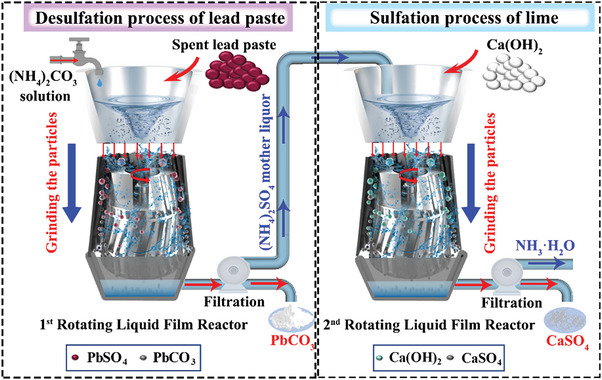
Schematic diagram of a new ultra‐rapid desulfation process of spent lead paste and sulfation process of lime via newly designed rotating liquid film reactors (RLFRs).

Herein, we propose a new ultra‐fast recyclable desulfation process of spent lead paste via the rotating liquid film reactor (RLFR) to grind the surface of particles and enhance the mass transfer process (**Scheme** **1**). Experimental and kinetic calculations manifest that the new reactor greatly promotes the removal process of desulfation products from the surface of unreacted PbSO_4_ particles and strengthens the mass transfer effect through the collision grinding effect of liquids and particles generated by the directional high‐speed rotation process in the reaction process. The new processes of desulfation of PbSO_4_ and sulfation of Ca(OH)_2_ can be instantly completed in 10 and 30 s, respectively, which significantly enhance reaction speeds, conversion efficiencies, and space‐time yield, achieving a directional, continuous, and sustainable technology, guiding a clean revolutionary improvement on the treatment of lead industry and recycling of the other related solid waste resources.

## 2 Results and Discussion

### 2.1 The Working Principle of the Rotating Liquid Film Reactor Intensification Process

In this case, the RLFR is proposed as a new intensification device to rapidly collide raw materials efficiently by instantaneous solid–liquid surface renewal with a greatly increased mass transfer rate to break through the diffusion‐controlled chemical reaction process. During the working process of the RLFR, the gap between the rotor and the stator is fine‐tuned, and the materials collide and grind in high‐speed movement through the gap, thereby the materials can achieve four purposes of mixing, dispersing, pulverizing, and reacting in the RLFR, and finally, fine productions are obtained.^[^ ^30^, ^31^ ^]^ Compared with the low reaction efficiency, long reaction time, and discontinuous reaction caused by the reaction products coated on the unreacted raw materials in the traditional reactor, 1) the RLFR possesses the tremendous shear force generated by the rotor‐stator system, which can make the prepared materials have the characteristics of small average particle size and narrow particle size distribution, greatly enhancing the microscopic mixing and mass transfer process. 2) The RLFR with the small confinement space and high rotation speed, makes raw material and liquid rapidly pass through the gap between the high‐speed rotor and stator under the action of extrusion, which causes the continuously exposed new surfaces to contact with the desulfation agent, thus greatly shortening the desulfation time and improving the reaction efficiency. Thus, the new device instantly generates a large grinding force and enough transformation chance among material particles, leading to the forced renewal material surface and the instantaneous accomplishment of the reaction.

### 2.2 Dual Intensification Processes of Desulfation of Lead Paste and Sulfation of Lime

**Figure 1:**
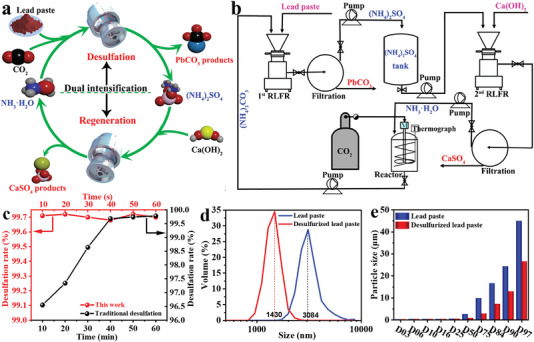
a) Schematic illustration of a new ultra‐fast recyclable and value‐added desulfation method for spent lead paste via dual RLFRs intensification processes. b) The flow diagram of the intensification desulfation and lime sulfation process via dual RLFRs intensification processes. c) Comparison of traditional desulfation and intensification desulfation based on a new RLFR. d) Particle size distribution of spent lead paste and desulfation lead paste. e) Particle size of spent lead paste and desulfation lead paste.

Thermodynamically, the desulfation reaction (1) of (NH_4_)_2_CO_3_ and PbSO_4_ and the sulfation reaction of Ca(OH)_2_ and (NH_4_)_2_SO_4_ are spontaneous processes with the reduced Gibbs free energy value (△_r_ *G*_m_) according to Lange's Handbook of Chemistry.^[^ ^32^ ^]^ Based on the solubility product (*K*_sp_) values of PbSO_4_ (*K*_sp_ = 1.6 × 10^−8^), PbCO_3_ (*K*_sp_ = 7.4 × 10^−14^), Ca(OH)_2_ (*K*_sp_ = 5.5 × 10^−6^), and CaSO_4_ (*K*_sp_ = 9.1 × 10^−6^), it is known that these two solid–liquid phase reactions are ways in which one insoluble compound generates another more insoluble compound. Due to the low solubility of these two solid–liquid phase reactions, resulting in a sluggish kinetics process, it is a great primary challenge to accelerate the actual reaction speed and conversion rate during the reaction. In this paper, **Figure** **1**a,b shows the desulfation process of spent lead paste and (NH_4_)_2_CO_3_ and the sulfidation process of (NH_4_)_2_SO_4_ mother liquor and Ca(OH)_2_ by the intensification process of RLFR, which improves the inherent shortcomings of traditional reactors, revealing the versatility for different types of reactions.

(1) $$\begin{array}{ccc} & & {\mathrm{PbSO}}_{4}+{\left({\mathrm{NH}}_{4}\right)}_{2}{\mathrm{CO}}_{3}\to {\mathrm{PbCO}}_{3}+{\left({\mathrm{NH}}_{4}\right)}_{2}{\mathrm{SO}}_{4}\Delta_{r}\;G_{m}^{\theta }\\ & & \;=-27.6{\;\mathrm{kJ}\;\mathrm{mol}}^{-1} \end{array}$$

(2) $$\begin{array}{ccc} & & \mathrm{Ca}{\left(\mathrm{OH}\right)}_{2}+{\left({\mathrm{NH}}_{4}\right)}_{2}{\mathrm{SO}}_{4}\to {\mathrm{CaSO}}_{4}+2{\mathrm{NH}}_{3}\cdot H_{2}{O\;\Delta }_{r}\;G_{m}^{\theta }\\ & & \;=-30.8{\;\mathrm{kJ}\;\mathrm{mol}}^{-1} \end{array}$$

#### 2.2.1 Intensification Desulfation Process of Spent Lead Paste and Ammonium Carbonate

Figure 1c shows the desulfation process of spent lead paste and ammonium carbonate in the three‐necked flask and RLFR, respectively. The desulfation process in the traditional stirred reactor reaches a desulfation rate of 99.6% after 40 min. In the traditional stirred reactor, the as‐obtained PbCO_3_ product will tensely cover the surface of the unreacted PbSO_4_ due to its low solubility, leading to a slowly degraded reaction with a low desulfation rate. In the intensification desulfation process, it endows a high desulfation rate of 98.8% in 5 s and 99.7% after 10 s. Figure 1d,e exhibits that desulfurized lead paste is well ground and its average particle size is reduced from 3.08 to 1.43 µm via RLFR by the laser particle size analyzer. The above results reveal that the mixture of spent lead paste and (NH_4_)_2_CO_3_ solution is instantly ground and pulverized through multiple functions of high‐speed shearing, squeezing, and impacting under vertical gravity, which promotes the forced renewed of the surface of spent lead paste and continuously exposes a new surface to contact with the desulfurizer, greatly shortening the desulfation time and improving the reaction efficiency.

**Figure 2:**
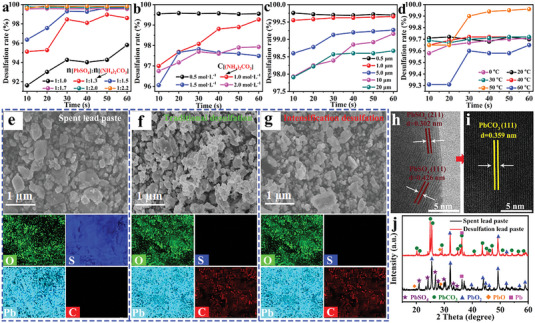
Factors affecting desulfation rates of spent lead paste: a) different molar ratios of (NH_4_)_2_CO_3_ to PbSO_4_, b) (NH_4_)_2_CO_3_ concentrations, c) the distances between the stator and the rotor, and d) reaction temperatures. Characterization of spent lead paste and desulfation lead paste: SEM and the corresponding element mapping images of e) spent lead paste, f) traditional desulfation lead paste, and g) desulfation lead paste by the new intensification desulfation process based on the RLFR. HR‐TEM image of h) spent lead paste and i) desulfation lead paste. j) PXRD patterns of spent lead paste and desulfation lead paste.

To deeply reveal the intensification desulfation process via the RLFR, we have systematically studied the effects of reaction times, different molar ratios of (NH_4_)_2_CO_3_ to PbSO_4_, (NH_4_)_2_CO_3_ concentrations, the distance between the stator and the rotor, and reaction temperatures on the desulfation rate. With the increase of molar ratios of PbSO_4_ to (NH_4_)_2_CO_3_ from 1:1 to 1:1.7, the desulfation rate increases from 91.6% to 99.6% at 10 s (**Figure** **2**a), suggesting a significant increase in the desulfation rate. Meanwhile, the desulfation rate stably maintains up to 99.7% with the increase of the molar ratio of PbSO_4_ to (NH_4_)_2_CO_3_ to 1:2.2, delivering that an appropriate molar ratio can accelerate the desulfation process and the reaction rate. Figure 2b displays the effect of various concentrations of (NH_4_)_2_CO_3_ on the desulfation rate. It confirms that the increase in concentration and viscosity of (NH_4_)_2_CO_3_ solutions, resulted in a reduction of the desulfation rate. Figure 2c reflects the influence of the distance between the stator and rotor on the desulfation rate. When the distance between the stator and rotor is larger than the particle size of reactants, the RLFR does not achieve sufficient grinding and shearing effects on the materials, resulting in a decreased desulfation rate. Figure 2d shows that the desulfation rate gradually increases as the temperature is from 0 to 50 °C. However, when the temperature is higher than 60 °C, the desulfation rate begins to decrease, which is attributable to the partial decomposition of (NH_4_)_2_CO_3_ at 60 °C. Benefitting from its high‐speed grinding and enhanced liquid dispersion of RLFR, it achieves up to 240 times desulfation speed than traditional reactors at room temperature, demonstrating a remarkable process intensification effect. So the optimal desulfation conditions of this process are as below: a molar ratio of PbSO_4_/(NH_4_)_2_CO_3_ is 1:1.7, the (NH_4_)_2_CO_3_ concentration is 0.5 mol L^−1^, the reaction time is 10 s, a distance between the stator and rotor is 0.5 µm, and the desulfation efficiency of spent lead paste is as high as 99.7% under room temperature.

Figure 2e–g shows the morphologies by SEM and the corresponding element mapping images of spent lead paste, and desulfation lead paste by the traditional stirred reactor and the RLFR under the optimal condition of desulfation. These results reveal that the particle size of desulfation lead paste by the RLFR is much smaller among the three samples, and it presents the main elements of Pb, O, and C without S elements, indicating that the PbSO_4_ of spent lead paste has been completely converted into PbCO_3_. HR‐TEM images further prove that PbSO_4_ has fully transformed into PbCO_3_, confirming the high efficiency of the new desulfation process (Figure 2h‐i). The main components of the desulfation lead paste are PbCO_3_, PbO_2_, PbO, and Pb demonstrated by the PXRD pattern in Figure 2j, further indicating that the PbSO_4_ in the spent lead paste is completely converted into PbCO_3_.

#### 2.2.2 The Determination of Kinetic Analyses for the Intensification Desulfation Process

**Figure 3:**
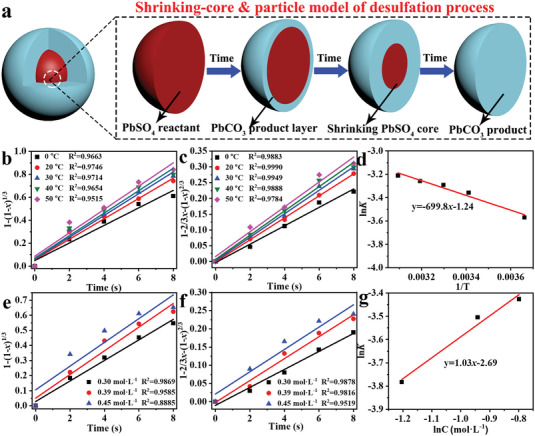
Dynamic analysis of the desulfation process. a) Schematics of shrinking‐core and particle model for the desulfation mechanism. Curves fitting of b) chemical reaction and c) internal diffusion governing equations at different temperatures. d) The relationship between 1/*T* and ln*K*. Curves fitting of e) chemical reaction and f) internal diffusion governing equations at different concentrations. g) The relationship between ln*K* and ln*C*.

The reaction between PbSO_4_ and (NH_4_)_2_CO_3_ is a solid–liquid phase reaction, with most of the reaction occurring at the interface. It is assumed that the desulfation reaction of PbSO_4_ reacts from the outside to the inside. Firstly, when the reaction time progresses, reaction (1) continuously shrinks to the internal solid and generates a new PbCO_3_ product layer, accompanied by gradual thickening and encapsulation of the PbCO_3_ product on the surface of solid raw material, leading to a slowing down of the reaction and a shrinking core of unreacted raw material solid. This reaction can be characterized by the shrinking core model of the PbSO_4_ desulfation (**Figure** **3**a).^[^ ^33^, ^34^, ^35^ ^]^

If the reaction process is controlled by a chemical reaction, the reaction kinetic equation can be written as^[^ ^36^ ^]^

(3) $${\left[1-{\left(1-x\right)}^{1/3}\;\right]}^{N}=K_{C}\;t$$

If the reaction process is controlled by internal diffusion, the reaction kinetic equation can be represented as^[^ ^30^ ^]^

(4) $${\left[1-\frac{2}{3}x-{\left(1-x\right)}^{2/3}\;\right]}^{N}=K_{D}\;t$$

where *K*_C_ is the rate constant of the hydration reaction. *K*_D_ is the diffusion rate constant. *N* is the reaction order, here *N* = 1. *x* is the desulfation rate; *t* is the reaction time (s).

According to Arrhenius formula, the relationship between temperature and reaction rate constant *K* is^[^ ^37^ ^]^

(5) $$In\;\left(K\right)=\;In\left(A\right)-\frac{E_{a}}{RT}$$

where *T* is the thermodynamic temperature (K). *K* is the reaction rate constant at temperature; *A* is the pre‐exponential factor; *R* is the thermodynamic gas constant (8.314 J mol^−1^ K^−1^). *E*_a_ is the apparent activation energy (kJ mol^−1^).

Figure 3b,c and Table S2, Supporting Information, show the relationship between reaction time and desulfation rate at different temperatures. The experimental results show that the desulfation rates of spent lead pastes reach 94.1%, 98.3%, 99%, 99.2%, and 99.6% at 8 s when the temperature increases from 0 to 50 °C, respectively, indicating the RLFR greatly facilitates the desulfation process, 50 °C is best reaction temperature. It can be seen that the linear relationship (*R* values) between 1−(2/3)*x*−(1−*x*)^2/3^ and reaction time (*t*) is significantly better than that between 1−(1−*x*)^1/3^ and *t* under different reaction temperatures, further indicating that the reaction between PbSO_4_ and (NH_4_)_2_CO_3_ is dominated by the internal diffusion. Thus, the *K*_D_ of Equation (4) obtained by fitting from Figure 3c is the diffusion reaction rate constant of PbSO_4_ at different temperatures. In addition, Figure 3d shows that the *E*_a_ value of the Arrhenius formula (5) is 5.82 kJ mol^−1^ by analyzing the linear relationship between 1/*T* and In*K*. Because the *E*_a_ value is not in the range of 30–85 kJ mol^−1^, it is inferred that the process is not controlled by surface chemical reactions but by internal diffusion.

Furthermore, the relationship between the reaction temperatures (*T*) and *K* can be obtained from the Arrhenius equation, as shown in the following Equation (6)

(6) $$\frac{K\left(T_{2}\right)}{K\left(T_{1}\right)}=e^{\frac{-E_{a}\left(T_{1}-T_{2}\right)}{RT_{1}T_{2}}}$$

For example, when *E*_a_ = 4.20 kJ mol^−1^, *T*_1_ = 293.15 K, and *T*_2_ = 303.15 K are substituted into Equation (6)

(7) $$\frac{K\left(303.15\;K\right)}{K\left(293.15\;K\right)}=e^{\frac{-5.82\;\times \;10^{3}\;\times \;\left(293.15\;-\;303.15\right)}{8.314\;\times \;293.15\;\times \;303.15}}\;=\;1.082$$

When *T*_1_ = 313.15 K and *T*_2_ = 323.15 K are substituted into Equation (6)

(8) $$\frac{K\left(323.15\;K\right)}{K\left(313.15\;K\right)}=e^{\frac{-5.82\;\times \;10^{3}\;\times \;\left(313.15-323.15\right)}{8.314\;\times \;313.15\;\times \;323.15}}\;=\;1.072$$

According to the above calculation results, the *K* values increase slowly with the increase of *T*. It is the low *E*_a_ value that leads the reaction to be insensitive to temperature changes.^[^ ^38^ ^]^ Therefore, the introduction of new catalysts can reduce the *E*_a_ values of this desulfation reaction and the influence of temperature on the desulfation rate, which will become the focus of follow‐up research.

The effect of different concentrations of (NH_4_)_2_CO_3_ on the desulfation reaction rate is further evaluated as shown in Figure 3e,f. The value of *R* from the fitting curves exhibits that the desulfation process is still dominated by internal diffusion while changing (NH_4_)_2_CO_3_ concentrations. The slope of 1.03 in the linear regression relationship between ln*K* and ln*C* is the apparent reaction order of the desulfation reaction, indicating that the reaction rate is proportional to the concentration to the power of 1.03^[^ ^39^ ^]^ (Figure 3g). Therefore, the rate equation for this reaction is

(9) $$-\;\frac{dC_{A}}{dt}=K_{A}\;C_{A}^{1.03}$$

When the optimal molar ratio of (NH_4_)_2_CO_3_ to PbSO_4_ is 1.7:1, the desulfation rate reaches 99.7% after 10 s and the consumption of (NH_4_)_2_CO_3_ and PbSO_4_ 0.2991 mol involved in the reaction (1). Based on the above kinetic process analysis, the desulfation process of spent lead paste is still controlled by internal diffusion. Therefore, the intensification effect of shearing, mechanical grinding, and ultrasonic crushing is the key to improving the desulfation rate of lead paste. It explains the great contribution of RLFR to enhance the conversion speed and desulfation rate of lead paste and also indicates that the desulfation conversion rate of lead paste is not sensitive to changes in reaction temperature and (NH_4_)_2_CO_3_ concentration.

#### 2.2.3 Intensification Sulfation Process of Lime and Ammonium Sulfate

**Figure 4:**
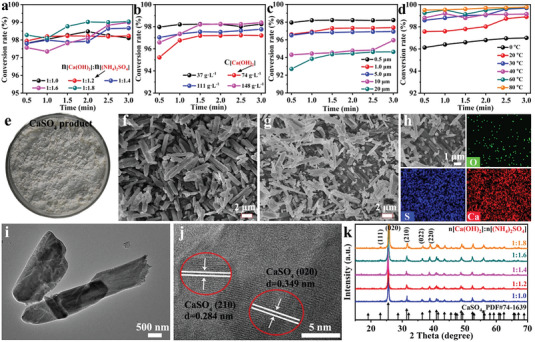
Factors affecting conversion rates of Ca(OH)_2_: a) molar ratios of Ca(OH)_2_ to (NH_4_)_2_SO_4_ at different reaction times, b) Ca(OH)_2_ concentrations, c) the distances between the stator and the rotor, and d) reaction temperatures. Characterization of CaSO_4_ products by the new intensification sulfation process based on the RLFR: e) Photo of CaSO_4_ product; SEM images of CaSO_4_ product by f) traditional lime sulfation and g) new lime‐intensification sulfation process based on the RLFR. h) Element mapping and i,j) TEM images of CaSO_4_ product; k) PXRD patterns of CaSO_4_ product.

The reaction of Ca(OH)_2_ and (NH_4_)_2_SO_4_ regenerates NH_3_·H_2_O solution and CaSO_4_, it found that formed CaSO_4_ was easily covered on the unreacted Ca(OH)_2_, resulting in the slow reaction rate and a low conversion rate.^[^ ^25^ ^]^ In this work, RLFR is introduced to enhance the conversion efficiency of Ca(OH)_2_ by systematically studying the effects of reaction times, different molar ratios of Ca(OH)_2_ to (NH_4_)_2_SO_4_, Ca(OH)_2_ concentrations, the distance between the stator and the rotor, and reaction temperatures on the sulfation rate. With the increase of molar ratios of Ca(OH)_2_ to (NH_4_)_2_SO_4_ from 1:1 to 1:1.8, the sulfation rate increases from 97.8% to 98.3% at 30 s (**Figure** **4**a). Meanwhile, different molar ratios of Ca(OH)_2_ to (NH_4_)_2_SO_4_ can achieve a conversion rate of 98–99% within 3 min, which corroborates that the molar ratio of raw materials has little effect on the conversion rate of Ca(OH)_2_. The remaining 1% is impurities from hydrated lime or tiny core particles. Figure 4b displays the effect of various concentrations of Ca(OH)_2_ on the sulfation rate, which suggests that the increase in concentration and viscosity of (NH_4_)_2_CO_3_ solutions results in a reduction of the sulfation rate.

In addition, the smaller distance between the stator rotors is beneficial to improve the sulfation rate of Ca(OH)_2_ from Figure 4c, which is because the RLFR plays a dominant role in the grinding and shearing effects by shortening the stator‐rotor distance, great grinding the particles at high speed and strengthening the dispersion of the liquid to promote the improvement of the conversion rate and the reaction speed. Figure 4d shows that the sulfation rate gradually increases from 96.1% to 99.5% at 30 s as the temperature is from 0 to 80 °C. However, the higher temperatures may cause the volatilization of the NH_3_·H_2_O solution and increase the heating energy consumption, 30–40 °C can be selected as the optimal reaction temperature in the actual reaction process. So the optimal conversion conditions of this process are as below: a molar ratio of Ca(OH)_2_/(NH_4_)_2_SO_4_ is 1:1.2, the Ca(OH)_2_ concentration is 37 g L^−1^, the reaction time is 30 s, a distance between the stator and rotor is 0.5 µm, the temperature is 30 °C, and the conversion efficiency of Ca(OH)_2_ is as high as 98.6%. This lime‐intensification sulfation process based on the RLFR has a Ca(OH)_2_ utilization rate as high as 99% and a reaction time as low as 30 s, providing the raw material basis for the regeneration of (NH_4_)_2_SO_4_ into NH_3_·H_2_O, which will be used for the desulfation of the next batch of spent lead paste after decomposition.

Figure 4e shows the photo of the obtained CaSO_4_ product through the lime sulfation intensification process by the RLFR device under optimal experimental conditions. The obtained CaSO_4_ has a finer rod‐like structure (Figure 4f,g). In addition, the corresponding element mapping images demonstrate the uniform distribution of S, O, and Ca elements in the overall structure of CaSO_4_ (Figure 4h). Two parallel fringe spacings of 0.284 and 0.349 nm are assigned to the (210) and (020) planes of CaSO_4_ (Figure 4i,j). The PXRD patterns of the sulfation product in Figure 4k also confirm the presence of pure CaSO_4_ (PDF#74‐1639), revealing the successful conversion of Ca(OH)_2_ into CaSO_4_. The residue Ca(OH)_2_ content has been titrated by the ethylenediaminetetraacetic acid (EDTA) titrationmethod through the dissolving of CaSO_4_ byproducts in excessive acetic acid (HAc, 0.5 mol L^−1^) solution, the results indicate the average Ca(OH)_2_ content is around 2%, revealing the conversion rates of Ca(OH)_2_ are about 98%.

### 2.3 Evaluation of Amplification Effect and the Recyclability of the Mother Liquor of RLFR

**Figure 5:**
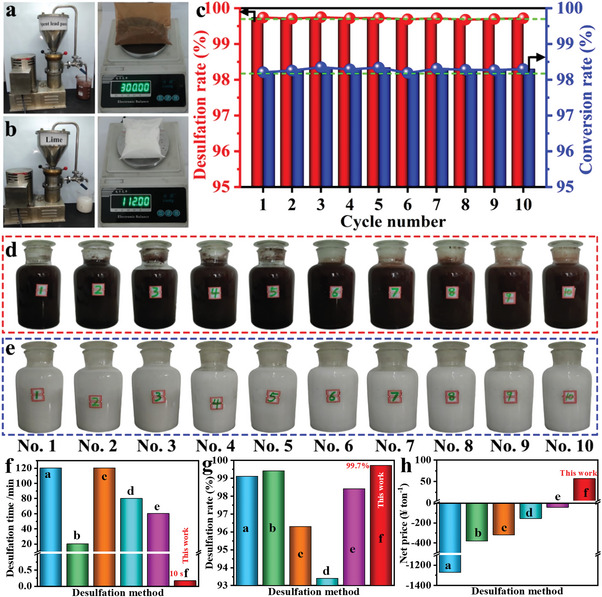
a) Photo of the RLFR device for intensification desulfation process of spent lead paste and raw material of spent lead paste (mass: 300 g); b) Photo of the RLFR device for intensification sulfation process of lime and raw material of lime (mass: 112 g); c) Desulfation rate and conversion rates of ten batches by the new dual intensification processes based on the RLFR; Photos of ten batches of d) desulfation lead paste and e) CaSO_4_ products by the new dual intensification processes based on the RLFR. Comparison of primary economic benefits in f) desulfation time, g) desulfation rate, and h) net price of different desulfation processes, a: Acetic acid‐sodium citrate desulfation, b: NaOH desulfation, c: Na_2_CO_3_ desulfation, d: NH_4_HCO_3_ desulfation, e: (NH_4_)_2_CO_3_ desulfation, f: This work.

In order to demonstrate the process amplification effect and the recyclability of the mother liquor of RLFR, we have taken 300 g per batch of spent lead paste and 112 g per batch of Ca(OH)_2_ as raw material and operated ten batches of continuous intensification desulfation and lime sulfation processes under optimal conditions (**Figure** **5**a,b). Figure 5c–e shows that the average desulfation rates are as high as 99.7% and the average conversion rates of Ca(OH)_2_ are as high as 98.2% in the ten batches, which confirms that the intensification desulfation process and the lime intensification sulfation process are stable and feasible, promoting and inspiring the green and low‐carbon treatment of other solid waste industries.

### 2.4 Evaluation of the Economic Benefits of the New Technology

To better evaluate the industrial implementation of the dual RFLR intensification desulfation process, we use 1 ton of spent lead paste as a unified starting point to calculate and compare the economic benefits of the traditional desulfation process and the new intensification desulfation process, as shown in Figure 5f–h and Table S3, Supporting Information. The new process can be completed in an extremely short reaction time of 10 s to achieve a desulfation rate of 99.7% (Figure 5f,g), which is 0.14–0.84% times the desulfation time in the existing desulfation process (20–120 min), significantly decreasing the reaction time and energy consumption of the desulfation process and promoting the green and low‐carbon development of the resource recovery industry. In addition, the reported traditional desulfation processes produce negative profits of −44.74 to −1270.16 ¥/ton due to the high cost of raw materials and evaporation cost of the sulfate solution by‐product, leading to the increase in the cost of the spent LABs recycling technologies. However, benefiting from the recycling of mother liquor and the high profit of gypsum by‐products, the new process can create a 55.99 ¥/ton primary economic benefit, achieving considerable economic and social benefits (Figure 5h).

## 3 Conclusion

In summary, we propose an ultra‐fast recyclable and value‐added desulfation method for spent lead paste via dual intensification processes in which two newly introduced RLFRs for the enhancement of the desulfation process of spent lead paste and sulfation process of lime and mother liquor. The systematic investigation of the dual intensification processes parameters and the simulation calculation in a mathematical model of the experimental results demonstrate that the intensification desulfation process of lead paste and sulfation process of lime are controlled by internal diffusion. RLFR device greatly promotes the improvement of the mass transfer process and reaction rate through high‐speed grinding, pulverizing, and enhanced liquid dispersion processes, which achieve the desulfation rate and conversion rate of the new process are 99.7% and 98.6% in 10 and 30 s, far higher than the 99.4% and 91.7% of the traditional process in 40 and 30 min, respectively. Moreover, ten batches of continuous scale‐up experiments also fully confirm the stability of the process. The new RLFR intensification process has brought huge technical and economic benefits in space‐time yield and raw material conversion rate, which can significantly reduce the investment cost and improve net profits of the recycling technologies, promoting the development of the lead industry and recycling of the other related solid waste resources.

## 4 Experimental Section

### Experimental Device

The experimental devices of desulfation of lead paste and sulfation of lime are shown in Figure S1, Supporting Information. The corresponding cross‐sectional structure of the RLFR main included the hopper, stator, rotor, electric motor, and body of RLFR.

### Intensification Desulfation Process for Spent Lead Paste

In the factor experiment, the desulfation process of 50 g spent lead paste (Chilwee Group) (Table S1, Supporting Information) and 70–150 mL (NH_4_)_2_CO_3_ solution was performed in an RLFR. The effects of different molar ratios of PbSO_4_ to (NH_4_)_2_CO_3_ in spent lead paste, reaction temperature, reaction time, (NH_4_)_2_CO_3_ concentration, and the gap between stator and rotor on desulfation rate were systematically studied.

### Intensification Sulfation Process for Lime

(NH_4_)_2_SO_4_ solution (the previous step) obtained by desulfation of spent lead paste reacted with 28 g Ca(OH)_2_ in the RLFR to produce ammonia water and calcium sulfate. The effects of different molar ratios of (NH_4_)_2_SO_4_ to Ca(OH)_2_, reaction temperature, reaction time, lime concentration, and the gap between stator and rotor on lime conversion rate were investigated. Compared with the new process, the traditional desulfation process used a three‐necked reactor with a motor stirrer in the laboratory to optimize the process parameters in the desulfation of PbSO_4_ and sulfation of Ca(OH)_2_.

### Regeneration of (NH_4_)_2_CO_3_ through the Desulfation and Sulfation Process

After the desulfation and sulfation process, the obtained (NH_4_)_2_SO4 reacted with Ca(OH)_2_ to generate ammonia solution and CaSO_4_. Then, the regenerated ammonia solution absorbed carbon dioxide that originated from the decomposition of desulfated lead paste to form (NH_4_)_2_CO_3_, which was recycled for the subsequent batch desulfation reaction. If the regeneration cannot be completed, the ammonia can be recovered from waste liquid by the simplified ammonia stripping technology. The regeneration ratio of (NH_4_)_2_CO_3_ is calculated by the following equation

(10) $$N\;\left(\%\right)=\frac{C_{1}V_{1}}{C_{2}V_{2}}\;\times 100\%$$

where *N* represents the regeneration ratio of (NH_4_)_2_CO_3_; *V*_1_ represents the volume of the regenerated ammonia absorbing carbon dioxide to produce ammonium carbonate; *C*_1_ (mol L^−1^) represents the concentration of (NH_4_)_2_CO_3_; *V*_2_ is the volume of (NH_4_)_2_SO_4_ produced by desulfation of spent lead paste; *C*_2_ (mol L^−1^) is the concentration of (NH_4_)_2_SO_4_.

## Conflict of Interest

The authors declare no conflict of interest.

## Supporting information

Supporting Information

## Data Availability

The data that support the findings of this study are available in the supplementary material of this article.
