# Supplementary material for: Ultra‐Fast Recyclable and Value‐Added Desulfation Method for Spent Lead Paste via Dual Intensification Processes
Source: Adv Sci (Weinh). 2023 Oct 22;10(34):2304863. doi: 10.1002/advs.202304863 (PMC10700223; doi:10.1002/advs.202304863)
Supplement: Supplementary file 1 — Supporting Information [file ADVS-10-2304863-s001.pdf]

ADVANCED SCIENCE
Open Access

Supporting Information

for *Adv. Sci.*, DOI 10.1002/advs.202304863

Ultra-Fast Recyclable and Value-Added Desulfation Method for Spent Lead Paste via Dual Intensification Processes

*Lulu Chai, Zhiyu Li, Keyu Wang, Xiaowei Liu, Shaozhen Dai, Xiaoguang Liu, Yanzhi Sun and Junqing Pan**

Supporting Information

Ultra-fast recyclable and value-added desulfation method for spent lead paste via dual intensification processes

*Lulu Chai, Zhiyu Li, Keyu Wang, Xiaowei Liu, Shaozhen Dai, Xiaoguang Liu, Yanzhi Sun, Junqing Pan**

1. Experimental part

1.1 Chemicals and materials

Ammonium carbonate ($(NH_4)_2CO_3$, AR, 30% NH_3) and ammonium sulfate ($(NH_4)_2SO_4$, ⩾99.0%) were purchased from China Fuchen (Tianjin) Chemical Reagent Co., Ltd. Disodium EDTA (EDTA-2Na, ⩾99.0%) was ordered from Tianjin Guangfu Technology Co., Ltd. Calcium hydroxide ($Ca(OH)_2$, ⩾95.0%) was purchased from China Xilong Science Co., Ltd. Deionized water was obtained by water purification system in the laboratory. The lead paste was provided by Anhui Cilwee Power Group. Firstly, spent lead paste samples were washed with deionized water, then dried in a vacuum oven at 120 ºC for 4 hours. Then it was crushed and sieved to obtain particles with a size of less than 100 μm.

1.2 Pretreatment of spent lead paste

The spent lead paste was obtained from the scrapped lead-acid batteries (LABs) provided by Anhui Chilwee Power group, China. Firstly, the scrapped LABs were automatically disassembled, crushed and sorted by an automatic splitter system to separate them into different components, such as lead paste, grids, sulfuric acid, separators, and plastics. Secondly, the sorted lead paste was washed several times with deionized water until the washing liquid was neutral, and then dried in an oven at 120 °C for 12 h. Finally, the lead paste was crushed with a crusher, and sieved with a 120-mesh sieve to remove the contained grids and fibers in the lead paste.

1.3 Material Characterization

The morphology and structure of the spent lead paste, the obtained highly pure $PbCO_3$, and $CaSO_4$ powder were analyzed with a scanning electron microscope (SEM, Japan Bruker Company, Model 8010) and a powder X-ray diffraction (PXRD, Japan Shimadzu Instruments Co., Ltd., XRD-6000). The samples were investigated with a thermogravimetric analyzer (TGA, Hitachi, STA7300) within 30-800 ^{o}C at a heating rate of 10 ^{o}C min^{-1}. Laser particle size analyzer (Malvern, MS2000) was used to test the size and distribution of particles. Moreover, an inductively coupled plasma atomic emission spectroscopy (ICP-AES, Thermo Fisher Technology China Co., Ltd., ICPA-6000) was used to determine the element concentrations of the samples.

1.4 The chemical composition of spent lead paste by chemical titration methods

1.4.1 The content measurement of PbO by the EDTA titration method

3 g lead paste was dissolved in a 5 wt% acetic acid solution (60 mL) for 30 min. The filtrate and filter residue were washed with 5 wt% acetic acid solution several times and separated by centrifugation.

Then 10 mL filtrate was placed in a conical flask, followed by ammonia ($NH_3 \cdot H_2O$) solution to adjust the pH of the filtrate to 5~6. Titration indicator was a mixture of 2 mL 20 wt% hexamethylenetetramine solution and 3 drops of 0.5 wt % xylenol orange. A 0.02 mol L^{-1} EDTA standard solution was employed for complexometric titration, and the result was calculated in the way:

$$\omega_{PbO} = \frac{25 \times c_{EDTA} \times v_{EDTA} \times M_{PbO}}{m} \times 100\%$$

where ω_{PbO} represents the mass fraction of the PbO component in the lead paste, c_{EDTA} and v_{EDTA} represent the concentration and volume of EDTA, M_{PbO} is the molar mass of PbO (223.2 g mol^{-1}), m stands for the total mass of the lead paste, respectively.

1.4.2 The content measurement of Pb the EDTA titration method

The above filter residue (step 1.1.1) was dissolved in 40 mL HNO_3 solution and stirred at 60 ^{o}C for 30 min. The filtrate and filter residue were washed with HNO_3 solution several times and separated by centrifugation. The newly obtained filtrate was analyzed by referring to (1.1.1) titration analysis.

$$\omega_{Pb} = \frac{25 \times c_{EDTA} \times v_{EDTA} \times M_{Pb}}{m} \times 100\%$$

Where ω_{Pb} is the mass fraction of Pb and M_{Pb} is the molar mass of Pb (207.2 g mol^{-1}).

1.4.3 The content measurement of PbO_2 the $KMnO_4$ titration method

The filter residue (step 1.1.2) was dissolved in a mixture of 40 mL HNO_3 and 5 mL H_2O_2 solution and stirred for 30 min. The filtrate and filter residue were washed with acetic acid solution several times and separated by centrifugation.

The newly obtained 10 mL sucked filtrate was placed in a conical flask and titrated with 0.1 mol L^{-1} calibrated $KMnO_4$ solution, which turned light red without fade in 30 s, recorded the volume of $KMnO_4$ solution (v_{KMnO4}). According to the above method, the blank experiment was conducted under the same conditions and recorded the volume of $KMnO_4$ solution (v_0).

$$\omega_{PbO_2} = \frac{5 \times c_{KMnO_4} \times (v_0 - v_{KMnO_4}) \times M_{PbO_2}}{m} \times 100\%$$

Where ω_{PbO2} represents the mass fraction of the PbO_2 in the lead paste, c_{KMnO4} and v_{KMnO4} stand for the concentration and volume of $KMnO_4$, v_0 is the volume of $KMnO_4$ solution required for the blank experiment, M_{PbO2} is the molar mass of PbO_2 (239.2 g mol^{-1}), respectively.

1.4.4 The content measurement of $PbSO_4$ the EDTA titration method

The filter residue obtained from the previous step 1.1.3 was dissolved in 20 wt% ammonium acetate solution, and boiled for 10 min at a heating mantle. After that, the reaction was naturally cooled down to room temperature and washed three times with deionized H_2O by centrifugation.

The newly obtained filtrate was analyzed by referring to the titration analysis in step (1.1.1).

$$\omega_{PbSO_4} = \frac{25 \times c_{EDTA} \times v_{EDTA} \times M_{PbSO_4}}{m_{Lead\ paste}} \times 100\%$$

Where ω_{PbSO4} represents the mass fraction of the $PbSO_4$ in the lead paste and M_{PbSO4} is the molar mass of $PbSO_4$ (303.27 g mol^{-1}).

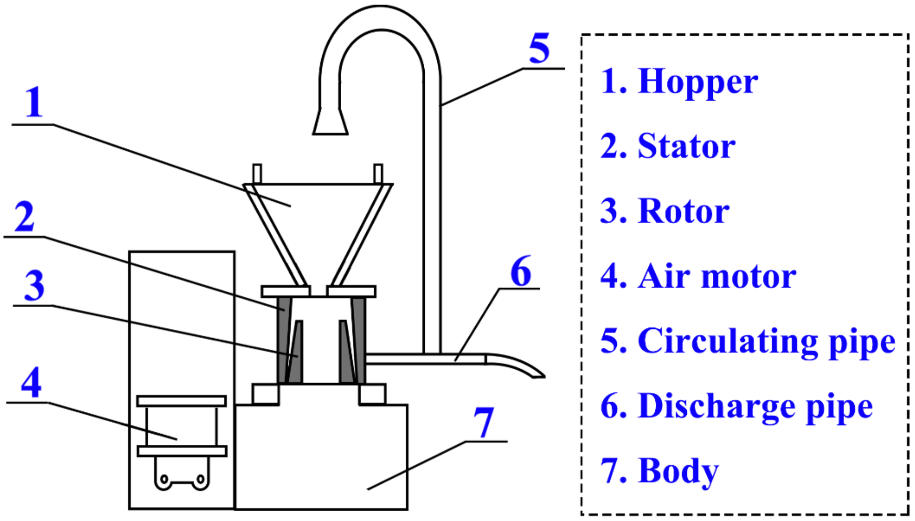

Figure S1. (a) Physical image and (b) cross-sectional view of rotating liquid film reactor (1. Hopper; 2. Stator; 3. Rotor; 4. Air motor; 5. Circulating pipe; 6. Discharge pipe; and 7. Body of rotating liquid film reactor).

Table S1. The chemical composition of lead paste by chemical titration methods.

Samples	$PbSO_4$ / wt%	PbO / wt%	PbO_2 / wt%	Pb / wt%
1	30.71	14.32	33.69	16.01
2	31.10	14.34	33.67	15.61
3	31.02	14.30	33.71	16.02
Average	30.94	14.32	33.69	15.88

Table S2. The desulfation rates of spent lead paste within 10 s at different reaction temperatures.

Desulfation rate	0 s	2 s	4 s	6 s	8 s	10 s
0 °C	0	55.0%	77.2%	90.3%	94.1%	99.6%
20 °C	0	65.4%	81.6%	93.0%	98.3%	99.71%
30 °C	0	68.9%	83.7%	95.6%	99.0%	99.69%
40 °C	0	70.1%	86.6%	97.0%	99.2%	99.69%
50 °C	0	76.4%	88.3%	98.1%	99.6%	99.65%

Table S3. Estimated net costs of different desulfation technologies.

Desulfation method	Single item	Dosage (kg)	Price (¥ ton^{-1})	Cost (¥ ton^{-1})	Net price (¥ ton^{-1})	Desulfation rate (%)	Desulfation time (min)	Ref.
Acetic acid–sodium citrate	$Na_3C_6H_5O_7{\cdot}2H_2O$	288.32	3900	-1124.45	-1270.16	99.1	120	[1]
	CH_3COOH	58.87	2000	-117.74				
	Na_2SO_4	139.25	300	41.77				
	MVR evaporation	-	-	-69.74				
NaOH-desulfation	NaOH	98.63	3600	-355.07	-382.78	99.4	20	[2]
	Na_2SO_4	140.09	300	42.03				
	MVR evaporation	-	-	-69.74				
Na_2CO_3-desulfation	Na_2CO_3	154.28	1890	-291.59	-319.98	96.3	120	[3]

	Na_2SO_4	137.84	300	41.35				
	MVR evaporation	-	-	-69.74				
**$(NH_4)_2CO_3$-desulfation**	$(NH_4)_2CO_3$	139.51	490	-68.36	-44.74	98.4	60	[4]
	$(NH_4)_2SO_4$	127.89	730	93.36				
	MVR evaporation	-	-	-69.74				
**NH_4HCO_3-desulfation**	NH_4HCO_3	146.14	1200	-175.37	-155.93	93.4	80	[4]
	$(NH_4)_2SO_4$	122.17	730	89.18				
	MVR evaporation	-	-	-69.74				
**This work**	$(NH_4)_2CO_3$	21.05	490	-10.31	55.99	99.7	0.167	
	CaO	46.09	170	-7.84				
	$CaSO_4$	132.39	560	74.14				

References

[1] X. Zhu, X. He, J. Yang, L. Gao, J. Liu, D. Yang, X. Sun, W. Zhang, Q. Wang, R. V. Kumar, *J. Hazard. Mater*. **2013**, *250-251*, 387.

[2] J. Pan, X. Zhang, Y. Sun, S. Song, W. Li, P. Wan, *Ind. Eng. Chem. Res.* **2016**, *55*, 2059.

[3] W. Yu, P. Zhang, J. Yang, M. Li, Y. Hu, S. Liang, J. Wang, S. Li, K. Xiao, H. Hou, J. Hu, R. V. Kumar, *J. Clean. Prod.* **2019**, *210*, 1534.

[4] X. Zhu, L. Li, X. Sun, D. Yang, L. Gao, J. Liu, R. V. Kumar, J. Yang, *Hydrometallurgy* **2012**, *117-118*, 24.
